# Adapting a Smartphone-Based Auricular Point Acupressure Self-Management Program for Rural Chronic Musculoskeletal Pain: Qualitative Study

**DOI:** 10.1177/11786329261472231

**Published:** 2026-07-24

**Authors:** Jungkyung Min, Peiyin Hung, Jane N. Bolin, Rupsikha Bora, Sarah Pace, Patricia Pitones, Hulin Wu, Jennifer Kawi

**Affiliations:** 1 Cizik School of Nursing, 12340 The University of Texas Health Science Center, Houston, TX, USA; 2 Arnold School of Public Health, 2629 University of South Carolina, Columbia, SC, USA; 3 School of Public Health, Texas A&M University Health Science Center, College Station, TX, USA; 4 College of Social Work, 2629 University of South Carolina, Columbia, SC, USA; 5 School of Public Health, The University of Texas Health Science Center, Houston, TX, USA

**Keywords:** chronic pain, auricular point acupressure, rural population, self-management, nonpharmacological treatment, complementary therapy

## Abstract

**Background:**

Chronic musculoskeletal pain is more prevalent in rural areas where access to pain interventions, including complementary approaches like Auricular Point Acupressure (APA), remains limited. APA is a non-invasive, needle-free approach, and its smartphone-based self-management delivery model has not been optimized for rural contexts.

**Objectives:**

To explore the perspectives of rural partners on adapting a smartphone-based Auricular Point Acupressure-Self-Management (APA-SM) program to better meet rural needs. Study objectives were to: (1) refine the APA-SM program for rural adults, (2) identify implementation barriers, (3) understand practice workflows and engagement, and (4) identify strategies to integrate the APA-SM program into rural pain care.

**Design:**

A qualitative study using semistructured focus group interviews.

**Methods:**

We conducted six semistructured focus group interviews with 24 participants who were purposively selected across South Carolina and Texas between October and December 2024. Data were analyzed thematically using an inductive approach following Braun and Clarke’s methodology. Ethical approval was obtained from a single Institutional Review Board. A limitation of the study was its regionally focused sample, which may hinder generalizability.

**Results:**

Four key themes emerged regarding partners’ perspectives on the APA-SM program: (1) building trust with rural communities, providing meaningful incentives, and having well-trained research staff; (2) communicating the value of APA and providing support through detailed information, motivational messaging, and guidance on integrating APA into daily routines; (3) ensuring accessibility and usability by providing educational materials in appropriate formats and addressing barriers related to technology gaps and communication needs; and (4) fostering partnerships with rural healthcare systems for effective implementation.

**Conclusion:**

These findings provide practical guidance for refining the APA-SM program to better fit the needs, barriers, and care contexts of rural communities.

## Introduction

Chronic pain, defined as pain persisting for 3 months or more, affected nearly 51.6 million adults in the United States in 2021—equivalent to one in four individuals.^1^ Chronic musculoskeletal pain (CMP), which affects the spine and joints, is a prevalent chronic pain condition in the United States and accounts for 5.8% of the national GDP in economic impact.^2^ Notably, rural populations experience a higher prevalence of CMP than urban areas and face additional challenges such as higher rates of disability and limited access to specialized care.^3,4^ Although the prevalence of CMP continues to rise, researchers have conducted limited studies on nonpharmacologic pain management interventions for underserved populations, particularly in rural communities.^5^ To reduce reliance on opioids for pain management, the U.S. Department of Health and Human Services recommends nonpharmacologic therapies as first-line treatment for chronic pain.^2,6^

Auricular point acupressure (APA) is a noninvasive, needle-free pain management technique based on acupuncture principles. It involves placing small vaccaria seed pellets on specific ear points corresponding to pain location in the body. Users are instructed to press the pellets at least three times per day, with each session lasting approximately 3 minutes, resulting in a total of 9 minutes of stimulation daily.^7,8^ Prior studies suggest that APA may be a feasible and acceptable nonpharmacologic approach for chronic pain self-management, with no reported adverse effects thus far.^7-13^ Existing studies, including pilot and feasibility trials, have reported acceptability, perceived usefulness, and preliminary signals of potential pain-related benefits, including improvements in pain, physical function, and medication use.^7,9,10,14-17^ A recently published fully-powered randomized controlled trial among older adults in an urban setting reported that APA, compared with an education control, improved pain and function, sustained at the 6-month follow-up.^7^ Experimental and neuroimaging studies suggest potential underlying mechanisms, including modulation of neural activity and inflammatory processes such as interleukin-1β, interleukin-2, as well as activation of brain regions implicated in somatosensory regulation.^6,18-21^

Many APA study participants in our previous clinical trials expressed a strong desire to continue APA treatment as part of their pain self-management after study completion.^7,10^ In response, we developed the APA-Self-Management (APA-SM) program, which included a smartphone app with instructional and demonstration videos. The app was initially developed and tested in prior studies involving primarily urban and metropolitan populations.^10,17^ In the present study, we further revised the existing APA-SM program for rural communities. For example, we added a Spanish-language version and more detailed seed-placement instructions.

Mobile health (mHealth) technologies offer scalable tools for chronic pain self-management through remote education and symptom monitoring, with evidence supporting their effectiveness in improving accessibility and self-efficacy.^22,23^ However, most mHealth programs are designed for urban or well-resourced users, with limited data on feasibility and adoption in rural contexts.^3^ Rural populations face compounding barriers to pain care, including geographic isolation, limited transportation, a shortage of pain specialists, and psychosocial differences in health beliefs and social contexts, that further limit access to nonpharmacologic pain management.^3,24,25^ A self-managed APA approach delivered via mHealth offers significant potential to overcome these systemic and geographical barriers. Yet, compared to the growing body of literature on APA in general populations, research on its application specifically in rural settings remains limited. To address this gap, we aimed to understand rural partners’ perspectives in adapting APA-SM into rural healthcare systems and communities.

### Purpose of the Present Study

This study aimed to explore the perspectives of rural partners in adapting and refining a smartphone-based APA-SM program to better meet the unique needs of rural populations. Specifically, this study sought to answer two guiding questions: (1) *How should we best refine (improve, adapt) the APA-SM program to meet the needs of rural populations?* (2) *How can we best integrate the APA-SM program into rural pain care?* Study findings were intended to achieve the following objectives: (1) refine the APA-SM program for rural adults, (2) identify barriers to implementation, (3) understand practice workflows and engagement, and (4) identify strategies for integration of the APA-SM program into rural pain management.

#### Ethical Considerations

This study was approved by the single Institutional Review Board at University of Texas Health Science Center at Houston (IRB No. HSC-SN-24-0182; approved April 2, 2024). All participants received an IRB-approved letter of information and provided consent before participation.

## Methods

### Study Design

This qualitative study employed six semistructured focus group interviews to capture diverse perspectives and contextual input from rural partners. A qualitative approach was appropriate for addressing the study’s purpose and guiding questions, as it enabled an in-depth exploration of participants’ viewpoints, perceived barriers, and recommendations for refining the APA-SM program for integration into rural pain care.^26^ We adhered to the Consolidated Criteria for Reporting Qualitative Research (COREQ) for transparency.^27^ The completed COREQ checklist is provided as Supplementary File 1.

### Participants and Recruitment

Participants were recruited through study flyers distributed by rural community partners (e.g., healthcare clinics, community health workers [CHWs]). Using purposive sampling, we targeted two stakeholder groups: (1) rural community partners serving rural areas, including primary care and specialty pain providers, healthcare staff or administrators, CHWs, policymakers, and significant others involved in rural pain care; and (2) individuals with CMP residing in rural ZIP Code Tabulation Areas or counties, as defined by the U.S. Department of Agriculture Rural-Urban Continuum Codes (RUCC), with eligibility limited to areas classified as RUCC 4 or greater.^28^ Healthcare providers were included as key stakeholders to help identify referral pathways and inform integration of the APA-SM program into rural clinical workflows. Inclusion criteria were adults aged 18 years or older belonging to any of the stakeholder groups. Individuals with CMP were required to report discomfort in the muscles, bones, joints, or contiguous connective tissues rated at least 4 out of 10 for at least 3 months, or pain on at least half of the days in the past 6 months. Significant others were excluded if they did not have a family or close relationship with an individual with CMP. Prospective participants were screened for eligibility by trained research staff prior to enrollment; those who met the inclusion criteria were enrolled in the study. Between October and December 2024, 27 participants were recruited from rural communities across South Carolina and Texas. Three participants did not attend their scheduled interview sessions (were unavailable and did not reschedule); therefore, 24 participants ultimately participated in the focus group interviews.

Participants were grouped by role into six focus group sessions. Group 1 included healthcare providers, CHWs, and significant others (n=6). Group 2 included healthcare administrators and policymakers across two sessions (n=3 and n=4, respectively). Group 3 included patients with CMP across three sessions: one Spanish-language group with primarily Spanish-speaking participants who are Hispanic (n=3), one group with bilingual Spanish and English-speaking participants who are Hispanic (n=4), and one group with English-speaking Black and White participants who are non-Hispanic (n=4). Language was retained as a grouping descriptor because it was considered relevant to cultural context and communication preferences that could shape participants’ perspectives on the APA-SM program. As compensation for their time and effort, each participant received a $30 e-gift card. Because this was a qualitative focus group study aimed at obtaining in-depth perspectives rather than estimating statistical effects, a formal power analysis was not conducted. An IRB-approved letter of information was provided to each participant prior to study participation. The letter of information contained necessary elements such as study purpose, description, procedures, risk, cost, incentive, privacy, confidentiality, and right to withdraw at any time.

### Data Collection

Six focus group interviews were conducted between October and December 2024 via Zoom. Since the interviews were carried out remotely, participants were able to schedule their interviews based on the dates, times, and settings (e.g., home) that were convenient, secure, and private for them. Each session lasted approximately 90 minutes, starting with an introduction and description of the APA-SM program, followed by time for participants’ questions about the program and then by the focus group interview. The primary data collection tool was a semistructured interview guide (Supplementary File 2), which was adapted from previously developed and pilot-tested questions used in the study team’s prior qualitative and mixed-methods studies on APA.^17,29,30^ Although the guide has not been pilot tested specifically with rural populations, the questions were reviewed in collaboration with rural community partners (e.g., Community Advisory Board [CAB]) to ensure relevance and clarity for the present study.

The approach using focus groups was selected to leverage group dynamics, encouraging participants to discuss topics they might not have considered in individual interviews, thereby yielding richer and more diverse data.^31-33^ To ensure privacy and data security, multiple safeguards were implemented (e.g., a waiting room was employed to control entry, only identified participants were allowed to join, interviewers and participants used pre-assigned participant ID numbers during the interviews for deidentification). Participant numbers allowed for internal study coding that is known only to designated and trained study team members. Interviews were recorded in audio only and securely stored on a password-protected institutional server accessible exclusively to the research team. The interviewer also recorded field notes during and immediately after each focus group to document contextual observations and group dynamics. Transcripts were summarized and returned to participants for review and comment. No feedback or requested changes were received. The themes were also shared and reviewed with the participants and the CAB.

The interviewer is a PhD-prepared faculty member with experience and training in conducting qualitative research and expertise in pain management and APA. Some members of the author team had prior involvement in APA research and were familiar with the design, implementation context, and earlier evaluation of APA-SM. This familiarity provided contextual understanding for interpreting participants’ accounts, but it also raised the possibility that prior assumptions could influence the analytic process. The team therefore practiced bracketing and integrated reflexive discussion throughout coding and theme development. Authors discussed their expectations at different stages of coding, questioned whether emerging themes were grounded in the data, and returned to the transcripts if interpretations were unclear. Particular attention was given to negative cases and accounts that did not align with the team’s initial expectations. Authors who had not been directly involved in the intervention also reviewed emerging themes and helped challenge interpretations that risked relying too heavily on prior knowledge. In this way, familiarity with APA-SM was treated as a resource for contextual understanding, while reflexive checks helped reduce the risk that prior assumptions would predetermine the findings. Rapport was established with the participants during study enrollment and informational sessions provided details regarding APA-SM.

### Data Analysis

The study team performed thematic analysis using an inductive approach to identify recurring themes related to participants’ perspectives on the APA-SM program.^34,35^ The analytic process was designed to ensure transparency, traceability, and rigor throughout all stages of data management and coding.^36^ All transcripts were verified against audio recordings and imported into ATLAS.ti (version 24.2.1; ATLAS.ti Scientific Software Development GmbH). Data were analyzed using a systematic and transparent process to ensure credibility and consistency of the findings.^37^

Initially, the study team read the transcripts multiple times to ensure a comprehensive understanding of the data and enhance familiarity with the content. One member of the study team generated initial codes relevant to the research questions to uncover patterns, which were cross-checked by another team member. The study team met regularly to discuss discrepancies, refine the codebook, and ensure consistency through reflexive discussions. Similar texts were grouped under the same code, whereas new codes were created for different meanings. These coded data were then categorized into recurring themes that reflected participants’ perspectives on the APA-SM program. A shared codebook was iteratively refined during analysis, and key decisions were documented to maintain transparency and consistency. Three members of the study team reviewed and refined the final themes until consensus was reached. During iterative analysis across focus groups, we determined that both code and meaning saturation, rather than formal group-specific saturation, were reached when no new codes emerged and no additional insights substantially changed the interpretation of participant perspectives.

#### Patient and Public Involvement

Patients were involved as participants in focus groups. Rural community partners (e.g., CAB) were consulted as to the design and conduct of the study. The CAB was convened by the Principal Investigators and met quarterly to provide input and guidance from a rural community perspective.

## Results

**Figure 1.**
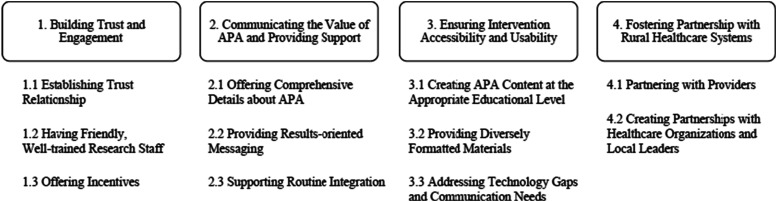
Thematic analysis for refinement of APA-SM for underserved rural populations

A total of 24 participants (6 focus groups) were included in the study. Of these, one-third were male (n=8, 33.3%), and nearly half identified as Hispanic or Latino (n=10, 41.7%). Participants ranged in age from 23 to 71 years (Table 1). Thematic analysis of the interviews revealed the following four themes: 1) building trust and engagement, 2) communicating the value of APA and providing support, 3) ensuring intervention accessibility and usability, and 4) fostering partnership with rural healthcare systems (Figure 1). The discussion of each theme is supported with direct quotes and illustrative examples.

**Table 1.** Participant Characteristics by Focus Group Type (N = 24)

Characteristic	Group 1 (healthcare providers, CHWs, and significant others, n = 6)	Group 2 (healthcare administrators, policymakers, and staff, n = 7)	Group 3 (patients with CMP, n = 11)	Total (N = 24)
**Age (years)**	Mean (SD): 54.86 (14.73)	Mean (SD): 54.86 (4.81)	Mean (SD): 45.55 (19.26)	Range: 23–71
**Gender, n (%)**				
Female	4 (66.7%)	6 (85.7%)	6 (54.5%)	16 (66.7%)
Male	2 (33.3%)	1 (14.3%)	5 (45.5%)	8 (33.3%)
**Race**				
White	4 (66.7%)	7 (100%)	8 (66.7%)	19 (79.1%)
Black	2 (33.3%)	0	3 (33.3%)	5 (20.9%)
**Ethnicity, n (%)**				
Hispanic or Latino	2 (33.3%)	2 (28.6%)	6 (54.5%)	10 (41.7%)
Not Hispanic or Latino	4 (66.7%)	5 (71.4%)	5 (45.5%)	14 (58.3%)
**Language used, n (%)**				
English	4 (66.7%)	6 (85.7%)	9 (81.8%)	19 (79.1%)
Spanish	2 (33.3%)	1 (14.3%)	2 (18.2%)	5 (20.9%)
**State, n (%)**				
South Carolina	3 (50%)	0	5 (45.5%)	8 (33.3%)
Texas	3 (50%)	7 (100%)	6 (54.5%)	16 (66.7%)

*Note.* Significant others were grouped with healthcare providers and CHWs to capture complementary perspectives on how APA-SM may be introduced and supported in rural pain.

### Theme 1. Building Trust and Engagement

This theme primarily addresses objective 1 (refining the APA-SM program) and objective 3 (understanding engagement) by showing that acceptability and uptake of APA-SM depend on trust-building strategies tailored to rural contexts. Across patient and healthcare administrator accounts, trust emerged as an important condition for rural engagement: patients linked trust to willingness to consider APA-SM, whereas administrators emphasized the role of trusted community organizations in introducing the program. This theme was divided into three subthemes, which are discussed below.

#### Subtheme 1.1: Establishing Trusting Relationships

Patient participants described trusting relationships as an important condition for considering and engaging with the APA-SM program. Trust was discussed as a way to reduce fear, increase credibility, and encourage openness to trying an unfamiliar self-management approach.

“*There’s a lot of fear … about various things, but when trust is established, it can make a big difference. Trust is built step by step by addressing our concerns and issues that make us hesitant, making sure where the information is coming from. That builds credibility” (Participant #27, Patient (Spanish)).*

*“For many Hispanics, trust comes from shared experiences. If someone says, 'Hey, I tried this, and it worked for me. Maybe you could try it too,' it makes people more open to trying something new” (Participant #4, Patient (Spanish)).*

Participants across stakeholder roles also identified trusted communication channels as important for introducing APA-SM in rural communities. Patient participants pointed to familiar local sources, such as radio stations and community announcements, whereas healthcare administrators emphasized partnerships with trusted local organizations and community venues, including Federally Qualified Health Centers (FQHCs), local clinics, churches, health departments, and other common gathering places.

*“I would say the radio station is probably really trusted along with other [community announcements]. If you want to get local news for rural places, that's really the only place you can hear it” (Participant #21, Patient (English)).*

*“Building strong relationships with local organizations is so important. For example, people are more likely to join if a trusted community group promotes the program rather than an outside researcher” (Participant #16, Healthcare Administrator).*

#### Subtheme 1.2: Having Friendly, Well-Trained Research Staff

Participants indicated they were more likely to participate in the program if they viewed the study team as reliable and respectful. This subtheme highlighted participants’ desire for friendly and professional research staff who could both educate and offer supportive guidance. Across stakeholder accounts, participants valued opportunities to share their concerns and personal stories with individuals who had similar backgrounds and life experiences. Building close relationships with instructors, such as CHWs, who understood their unique circumstances was identified as a key priority.

“*I think it would be really helpful to have a community health worker explain the program before participants get involved. Someone with a similar background can be an advocate, sharing their own experiences and challenges while also answering participants' questions” (Participant #16, Healthcare Administrator)*.

*“The staff should be friendly because if they are, participants will feel more comfortable opening up about their needs….” (Participant #7, Patient (English)).*

In addition, patients expressed a strong desire for accessible communication with the research team to receive timely feedback, ensure the accuracy of their self-management practices, and keep their providers informed about their progress. They emphasized the importance of regular check-ins and follow-up communication from the research team to support their engagement and adherence to the program.

#### Subtheme 1.3: Offering Incentives

The importance of financial incentives was frequently highlighted by both healthcare administrator and patient accounts as a strategy to support engagement, although their emphases differed. Healthcare administrator accounts stressed that financial support served as a reimbursement mechanism for organizations, acknowledging the significant resources required to implement the program effectively, including the time and involvement of healthcare providers and staff.

*“It primarily funds the research side, covering the person handling and managing the data. It doesn’t always directly fund someone in the healthcare organization but grant money does go to the healthcare entity to support the work” (Participant #10, Healthcare Administrator).*

Food and gasoline gift cards were commonly suggested as incentives. Additionally, patient participants recommended offering incremental incentives to mitigate potential declines in participation over time.

*“I remember in another study I did, it had six sessions, and the incentives started small, like $10. But as you continued, the rewards got bigger, and by the end, if you stayed with it, you received a significant amount. Something like that could keep people engaged” (Participant #21, Patient (English))*.

### Theme 2. Communicating the Value of APA and Providing Support

This theme speaks to objective 1 (refining the APA-SM program) and objective 2 (identifying barriers) by describing how tailored educational messages and motivational support can enhance understanding, reduce hesitation, and promote sustained engagement with the program. The second theme highlighted that educational efforts regarding APA-SM for rural populations should aim to enhance understanding of the program’s purpose. Participants believed that increased knowledge about the potential value and rationale for using APA could help boost participation. They also noted that rural populations may be more likely to engage with the program if initial motivation is provided. Participants emphasized the need for comprehensive informational support and emotional reassurance to encourage routine use of APA.

#### Subtheme 2.1: Offering Comprehensive Details About APA

When asked about effective strategies to disseminate the APA-SM program to rural populations, patient participants emphasized the need for clear, detailed information. Specifically, they wanted to understand what APA entails, how the intervention is intended to support pain self-management, the evidence behind it, and why they should participate. As many participants were unfamiliar with the APA-SM intervention, they expressed curiosity about its expected benefits, potential adverse effects, and noninvasive nature.

*“I think when something new comes up, people can feel unsure or even scared about it - like with COVID vaccines, a lot of people were nervous at first. A lot of hesitation comes from not understanding. Educating people clearly about how APA works could make a big difference” (Participant #15, Patient (English))*.

*“I’d like to know how long the little patch lasts, when it needs to be replaced, and if there are any side effects when stopping it” (Participant #6, Patient (English)).*

#### Subtheme 2.2: Providing Results-Oriented Messaging

This subtheme reflected participants’ openness to the APA-SM program and the value of motivational strategies, particularly those grounded in personal experiences or testimonials from trusted messengers, such as CHWs. Participants suggested that hearing relatable experiences from others who had tried the program could encourage participation and make APA-SM feel more approachable. Furthermore, some participants preferred small, in-person group sessions led by professionals to foster self-motivation and engagement. Healthcare provider and administrator accounts also emphasized the importance of framing APA-SM as a potentially useful additional self-management option for pain care.

*“In rural communities, it helps to have someone who has already participated in the program speak about their good feedback and challenges. They can make it more relatable” (Participant #13, Healthcare Provider).*

*“This could be a great tool for patients with opioid use disorder, giving them another resource to support their treatment. I think it would work well” (Participant #11, Healthcare Administrator).*

#### Subtheme 2.3: Supporting Routine Integration

This subtheme captured patient participants’ concerns about integrating the intervention into their daily routines. Because the intervention requires consistent seed placement and stimulation as part of the self-management protocol, patients expressed a desire to learn more about how to establish this intervention as part of their everyday habits.

“*I think scheduling is important. Maybe having a time scheduled for the APA session would help maintain consistency” (Participant #2, Patient (Spanish)).*

*“Adding it to my routine would make it easier to see results. My diet, exercises, and overall routines already require consistency. Including this at set times would fit well” (Participant #26, Patient (English))*.

### Theme 3. Ensuring Intervention Accessibility and Usability

This theme aligns closely with objective 2 (identifying barriers) and objective 3 (understanding engagement) by identifying practical and structural barriers such as health literacy, the need for diverse formats of educational materials, and access to technology that can affect rural participants’ ability to engage with the APA-SM program.

The third theme focused on specific barriers encountered by rural populations. Across stakeholder accounts, participants identified three key challenges, highlighting the importance of developing tailored strategies to help ensure effective implementation of the APA-SM program.

#### Subtheme 3.1: Creating APA Content at the Appropriate Educational Level

Healthcare administrator and patient accounts suggested that educational materials should be designed to target approximately third-grade reading level when possible. Informational materials should be simple, clear, well structured, and highly visual.

*“I think health literacy will play a crucial role in this project. From a health literacy perspective, the Hispanic populations we serve at [Clinic] face significant gaps and barriers. This is an important factor to consider moving forward” (Participant #10, Healthcare Administrator).*

*“Materials should be simple and clear. Easy to understand. Structured well for clients” (Participant #2, Patient (Spanish)).*

#### Subtheme 3.2: Providing Diversely Formatted Materials

Across healthcare administrator and patient accounts, participants emphasized the importance of tailoring the format of educational materials. Specifically, they suggested providing materials in multiple formats, including take-home manuals and Spanish/English bilingual versions. Participants also valued materials that can be easily shared in trusted community spaces, such as flyers, brochures, and videos.

“*I think oftentimes verbal instructions alone in the clinic setting aren’t enough. So, there will be needed written materials at an appropriate level to refresh their memory after leaving the clinic” (Participant #17, Healthcare Administrator)*.

*“If someone found a flyer at a trusted location, they would know that it's probably a good program” (Participant #2, Patient (Spanish)).*

#### Subtheme 3.3: Addressing Technology Gaps and Communication Needs

This subtheme highlighted communication challenges and limited access to adequate technology in rural areas that could affect engagement with the APA-SM program. Technology-related concerns were raised across multiple stakeholder accounts, including patients, healthcare administrators, and healthcare providers. Although participants noted that many individuals in rural communities have smartphones, patient and healthcare administrator accounts particularly emphasized barriers related to limited smartphone minutes, prepaid or “burner” phones, frequent phone number changes, and limited broadband access, while healthcare provider accounts highlighted poor cell service and restricted Wi-Fi access in rural communities.

“*And the rural community I grew up in, so I guess something else to consider going off on is the cell service or we only have a certain number of minutes that they pay for each month” (Participant #21, Patient (English))*.

*“Many rural residents rely on prepaid phones with limited minutes. So in such cases, we need to provide them with extra data or alternative ways to stay connected for long-term follow-up” (Participant #17, Healthcare Administrator).*

*“I just will agree that the changing of the phone numbers because they're not permanent numbers but they’re burner phones. That's been a challenge in the past. When we've worked with rural populations and had a lack of broadband Internet access” (Participant #19, Healthcare Administrator).*

To address these barriers, participants emphasized the need for practical technology and communication supports, including telehealth platforms, accessible spaces with computers and Wi-Fi, paper-based materials, and use of public buildings such as libraries for internet access.

*“Some areas have poor cell service, and many people don’t have unlimited data. They may need to rely on public Wi-Fi at places like libraries or McDonald's. So those are things to kind of ponder on when you're looking at the rural community” (Participant #13, Healthcare Provider).*

### Theme 4. Fostering Partnerships With Rural Healthcare Systems

This theme directly responds to objective 3 (understanding practice workflows and engagement) and objective 4 (identifying strategies for integrating the APA-SM program into rural pain care) by illustrating strategies for embedding the program within rural clinical settings and establishing sustainable community–provider collaborations.

The last theme highlighted the importance of building collaborative partnerships for recruitment and program implementation and longer-term integration. Healthcare provider and administrator accounts emphasized the need to minimize added workload and allocate adequate budget or resources to support staff time.

#### Subtheme 4.1: Partnering With Providers

This subtheme highlighted the importance of securing buy-in from primary care providers and pain specialists. A critical point for gaining healthcare providers’ buy-in is effectively communicating the core value and process of the intervention. Patient and healthcare administrator accounts framed provider buy-in in relation to clinic burden, emphasizing that APA-SM would need to be presented as supportive of rural care teams rather than as an added workload.

*“Clinics and organizations need to see how the APA-SM intervention can lighten their workload, not making it heavier from a patient care perspective” (Participant #3, Patient (English)).*

*“It's important for them to understand why this intervention matters and how it supports rural care teams, reducing their burden rather than adding to it” (Participant #10, Healthcare Administrator).*

Apart from primary care providers, partnering with other healthcare professionals can enhance opportunities for participant recruitment. When discussing effective approaches to recruiting individuals with CMP in rural communities, patient and healthcare provider accounts highlighted the value of partnering with other trusted professionals, including pharmacists, massage therapists, and chiropractors.

*“If chiropractors and massage therapists add these to their services, more people in the community would hear about it and give it a try” (Participant #22, Patient (English))*.

*“Please don’t forget about pharmacists. In rural areas, people trust their pharmacists and often ask them about treatments. If they’re informed about APA-SM, they can help spread the word” (Participant #13, Healthcare Provider).*

#### Subtheme 4.2: Creating Partnerships With Healthcare Organizations and Local Leaders

Healthcare provider and administrator accounts primarily discussed how the APA-SM program could be integrated into clinical workflows. Many expressed concerns that implementing the program might place an additional burden on existing staff unless proper incentives and support were allocated. Key issues raised during the interviews included: (1) the development of a scalable workflow for effective implementation, (2) the establishment of a memorandum of understanding (MOU) or agreement with the legal/risk management office, (3) budget considerations for facilities to extract and deidentify data, and (4) ongoing collaboration with healthcare partners and local leaders. Among these topics, funding was the most frequently cited concern. Administrator accounts highlighted the importance of ensuring adequate budget allocation, not only for patient-related aspects of the program but also to support healthcare providers involved in referrals and electronic health record (EHR) access.

*“We now have a system in place where, if we collaborate with a clinic, we create an MOU with a workforce agreement so our staff can access the EHR” (Participant #19, Healthcare Administrator).*

Last, a minor theme within this broader theme emerged from the focus group interviews, reflecting perceived facilitators for implementing the APA-SM program. Rural partners identified key elements that could support its integration into rural communities such as pain itself as a motivator, interest in APA as a home-based alternative, provider concerns about opioid use, and the perceived value of cost-conscious care.

*“I don’t like how pain medicine makes me feel. If this is an alternative, I’d try it, and I believe others would too” (Participant #3, Patient (English)).*

*“Health insurance plans, especially in rural areas, are really focused on cost-effective treatments that keep patients out of the hospital and reduce expensive care” (Participant #20, Policymaker).*

## Discussion

The aim of this study was to explore the perspectives of rural partners regarding a smartphone-based APA-SM program to refine its applicability for rural populations. Rural populations differ from urban cohorts in educational attainment, insurance status, and geography, often maintaining distinct expectations for healthcare.^38,39^ Our findings identify critical strategies for tailoring APA-SM to rural contexts, with emphasis on feasibility, perceived usefulness, accessibility, and implementation potential. This qualitative study was not designed to evaluate therapeutic efficacy or effectiveness.

In this study, rural partners identified strategies and barriers critical to optimizing a self-managed pain program for rural adults. APA-SM may help expand access to chronic pain education, self-management support, and culturally and linguistically responsive pain care in rural communities. Four key themes emerged from the analysis: (1) building trust and engagement, (2) communicating the value of APA and providing support, (3) ensuring intervention accessibility and usability, and (4) fostering partnerships with rural healthcare systems.

### Building Trust and Engagement

The first theme primarily addresses objective 1 (refining the APA-SM program) and objective 3 (understanding engagement). Establishing trusting relationships and offering phased incentives are essential for recruiting and retaining rural populations, consistent with prior research on virtual care settings.^3,40^ Trust is crucial for rural adults to feel comfortable sharing their concerns and ideas; experiencing trusting and caring relationships ultimately encourages them to adopt healthier behaviors.^41^ Additionally, friendly, approachable staff further facilitate engagement, mirroring findings by Brockman et al. (2023) and Tran et al. (2025), who highlighted the importance of trained staff in facilitating rural populations’ engagement in research.^42,43^

### Communicating the Value of APA and Providing Support

The second theme primarily addresses objective 1 (refining the APA-SM program) and objective 2 (identifying barriers). The need for comprehensive information about APA reflects findings from a comparable smartphone-based intervention for Hispanic populations, in which participants expressed the need for additional content.^44,45^ Motivational messages, including testimonials, positively influence participants’ active engagement in self-management programs.^46,47^ Additionally, incorporating APA into daily routines is a significant measure of participants’ engagement.^48^

### Ensuring Intervention Accessibility and Usability

The third theme primarily addresses objective 2 (identifying barriers) and objective 3 (understanding engagement). Educational materials must be available in diverse formats and tailored to appropriate educational levels. Prior research on rural populations emphasized that standardized materials may appear intimidating, highlighting the importance of simple, easy-to-read resources such as brochures, posters, and fact sheets.^49,50^ Employing CHWs with a deep understanding of rural communities’ language preferences is essential to ensure cultural sensitivity. The persistent digital divide in rural areas underscores the critical need for flexible delivery formats and ongoing follow-up.^51-53^ Clinic-based telehealth and partnerships with cellphone network providers could help bridge connectivity gaps support ongoing engagement.

### Fostering Partnerships With Rural Healthcare Systems

The fourth theme primarily addresses objective 3 (understanding practice workflows) and objective 4 (identifying strategies for integrating the APA-SM program into rural pain care). Partnerships with rural healthcare systems, trusted professionals, and local organizations appear essential for integrating APA-SM into rural pain care. Prior research has shown that local organizations, including retailers, churches, fitness centers, and rehabilitation facilities, can support rural health programs by assisting with outreach, advertising, and site identification.^54^ Sustaining these partnerships may require platforms that facilitate collaboration, knowledge sharing, and exchange of best practices.^55^ Collaborating with community organizations and local leaders enables researchers to develop culturally relevant interventions and tailored recruitment strategies.^40^ However, these collaborations often incur additional costs, such as compensation for community partners and investments in training and capacity-building. Accounting for these factors is essential to support sustainable implementation of community-driven health initiatives.

Overall, the study findings allowed us to understand how to best refine the APA-SM program for implementation in rural settings, identify implementation barriers that need to be addressed, and understand clinic practice workflows and concerns (Table 2). These provide valuable information to help with successfully implementing and sustaining the APA-SM program into rural pain care. The implications of our findings suggest that for this program to effectively serve rural populations, it must prioritize building trust within rural healthcare systems and community organizations, followed by effectively communicating the value of APA-SM. Addressing barriers specific to rural communities, such as providing appropriately tailored educational materials and ensuring alternative technology access options, is crucial for enhancing participation in the APA-SM program. Furthermore, engaging community partners necessitates careful consideration of financial and logistical factors, including workflows and fair compensation for their time and expertise as well as investments in training and capacity-building. By understanding participant perspectives, our research objectives were achieved, providing practical guidance for refining APA-SM and informing future efforts to integrate the program into rural pain care. If implemented and sustained in rural care settings, APA-SM may contribute to broader efforts to expand access to nonpharmacologic pain self-management options for underserved rural populations.

**Table 2.** Rural Partner-Informed Recommendations and Action Plans for Refinement of the APA-SM Program

Study theme	Rural partner perspectives based on study themes	Rural partner-informed recommendations	Action plans	Objectives addressed
1. Building Trust and Engagement	Trusted communication channels	Partner with churches, local radio station, health departments, community hubs; use CHWs and FQHCs as messengers.	Develop locally tailored outreach and referral materials for dissemination through trusted community messengers.	Barriers, Integration
	Participant incentives	Use phased and incremental incentives to sustain engagement and completion.	Use milestone-based incentives, including food or gas cards, to support engagement and retention.	Refinement, Integration
	Workforce training	Train CHWs and staff on APA basics, app navigation, and participant coaching.	Create standardized training and onboarding materials for community- and clinic-based personnel.	Refinement, Workflows
2. Communicating the Value of APA and Providing Support	APA education and communication	Clearly explain what APA is, how it works, expected benefits, and safety.	Add plain-language educational content, FAQs, and brief evidence-informed explanations to the app and accompanying materials.	Refinement, Barriers
	Motivational support and routine integration	Use testimonials, encouragement, and practical guidance to support routine use.	Integrate motivational messages, testimonials, and routine-support prompts into program content.	Refinement, Engagement
3. Ensuring Intervention Accessibility and Usability	Health literacy and materials	Create highly visual, step-by-step guides at approximately 3rd-grade level; simplify app and print content.	Revise app and print materials using plain language, visual supports, and simplified step-by-step instructions.	Refinement, Barriers
	Language accessibility	Provide bilingual (English/Spanish) materials and culturally resonant examples.	Add Spanish-language app content and bilingual print materials with culturally relevant examples.	Refinement, Barriers
	Technology access	Provide paper alternatives, leverage public Wi-Fi, and consider low-bandwidth app functions.	Offer paper-based materials and identify accessible community spaces with computers and Wi-Fi, such as libraries, to support participation.	Barriers, Integration
4. Fostering Partnerships with Rural Healthcare Systems	Workflow integration	Map clinic flow; insert brief APA-SM touchpoints; define responsible roles.	Develop workflow guidance for referrals, onboarding, kit distribution, and follow-up.	Workflows, Integration
	Data-sharing and governance	Establish MOUs; integrate referral and tracking fields in the EHR.	Formalize collaboration agreements and establish referral and tracking processes aligned with local systems.	Workflows, Integration

*Note.* All of these action plans in this table can be immediately implemented within the existing APA-SM program. However, a few action plans need significant administrative alignment and implementation research such as workflow and EHR integration as well as collaboration agreements and clinic processes to fully embed APA-SM within healthcare systems.

### Limitations

This study has some limitations. First, non-probability purposive sampling was used, which may introduce participation bias. Potential participants were identified and referred by existing community partners, which may have favored individuals who were more motivated or engaged in health-related activities. Results may have differed if referrals had come from non-partner sources. Second, generalizability and transferability may be limited because the data were collected from 24 participants across one southern state (Texas) and one southeast state (South Carolina). Although the recruitment and engagement of Hispanic participants was an important strength, particularly given their underrepresentation in pain research and their substantial presence in Texas, the sample may not fully reflect the diversity of rural populations across the United States, particularly in regions with different sociodemographic or healthcare characteristics. Because rural communities vary substantially by region in population composition, cultural and language needs, broadband access, transportation barriers, provider availability, clinic workflows, and community partnership structures, the findings may be most transferable to rural settings with similar southern/southeast, community-based, and healthcare access contexts. Future studies should include broader geographic representation and greater demographic diversity to strengthen the generalizability and transferability of findings. Another limitation is that the semistructured interview guide was not pilot tested among rural populations. Lastly, power analysis was not conducted because this was a qualitative study; however, sample adequacy was supported by methodological guidance for focus groups and the achievement of thematic saturation.^56^

## Future Directions and Recommendations

### Areas for Further Research and Investigation

Future research is needed to evaluate the long-term impact and scalability of the APA-SM program in rural communities. Longitudinal studies examining pain outcomes, adherence, and pain care utilization can clarify the program’s sustained effectiveness and contribution to reducing chronic pain disparities. Investigating the intersectionality of rural barriers including geographic isolation, broadband access, socioeconomic disadvantages, and cultural factors is essential for refining APA-SM to fit diverse populations. Future clinical trials should also assess how telehealth infrastructure can extend APA-SM delivery, for example by embedding APA coaching, troubleshooting, and follow-up into virtual primary-care or behavioral-health visits. Furthermore, integrating the APA-SM app within existing telehealth platforms could enable remote symptom monitoring, clinician feedback, and automated appointment reminders, ensuring continuity of care while minimizing travel burdens. Finally, community-driven research approaches should continue to empower rural partners to shape program content, incentive models, and culturally responsive materials. Collaboration with local organizations and CHWs can promote cultural relevance, trust, and long-term sustainability.

### Recommendations for Scaling and Integration

System-level alignment is critical for scalability. Reimbursement policies that recognize nonpharmacologic pain interventions, along with adequate budgeting for staff time and workforce training, can support sustained delivery. The long-term sustainability of the APA-SM program can be strengthened by public-private partnerships, which have been shown to help bridge rural health infrastructure gaps by aligning public equity goals with private-sector digital innovation.^57,58^ Healthcare institutions should also prioritize embedding APA-SM into primary care workflows, including brief visit-based touchpoints and EHR integration for referrals and tracking. Establishing MOUs with rural clinics can formalize shared responsibilities and data-use agreements.

Training and capacity building remain central. Clinicians, CHWs, and administrative staff should receive education on APA-SM fundamentals, patient communication, and digital health facilitation. Partner-informed implementation recommendations include: promoting the program through trusted community channels, providing plain-language and third grade-level bilingual materials, offering incremental incentives to sustain engagement, and addressing technology barriers through paper alternatives and public Wi-Fi access points. Collaborative learning platforms across institutions and communities can support mutual learning, sharing of evaluation outcomes, and strengthening adaptation. Continuous evaluation of these multi-level strategies will advance equitable, sustainable, and scalable integration of APA-SM into rural pain care.

## Conclusion

Understanding the perspectives of rural partners on APA-SM was vital to shaping the foundation and refinement of this intervention program. The findings from this study can guide researchers and healthcare professionals in helping to ensure that smartphone-based solutions and complementary modalities for managing CMP are accessible, contextually appropriate, and sustainable, thereby promoting equitable pain management in rural communities.

## Supplemental Material

Supplemental material - Adapting a Smartphone-Based Auricular Point Acupressure Self-Management Program for Rural Chronic Musculoskeletal Pain: Qualitative Study Supplemental material for Adapting a Smartphone-Based Auricular Point Acupressure Self-Management Program for Rural Chronic Musculoskeletal Pain: Qualitative Study by Jungkyung Min, Peiyin Hung, Jane N. Bolin, Rupsikha Bora, Sarah Pace, Patricia Pitones, Hulin Wu, Jennifer Kawi in Health Services Insights

Supplemental material - Adapting a Smartphone-Based Auricular Point Acupressure Self-Management Program for Rural Chronic Musculoskeletal Pain: Qualitative Study Supplemental material for Adapting a Smartphone-Based Auricular Point Acupressure Self-Management Program for Rural Chronic Musculoskeletal Pain: Qualitative Study by Jungkyung Min, Peiyin Hung, Jane N. Bolin, Rupsikha Bora, Sarah Pace, Patricia Pitones, Hulin Wu, Jennifer Kawi in Health Services Insights

## Data Availability

In accordance with the NIH HEAL Initiative Public Access and Data Sharing Policy, de-identified individual participant data and associated documentation (e.g., data dictionary, and metadata) are shared through the HEAL Data Platform.*
