# Supplementary material for: Adapting a Smartphone-Based Auricular Point Acupressure Self-Management Program for Rural Chronic Musculoskeletal Pain: Qualitative Study
Source: Health Serv Insights. 2026 Jul 24;19:11786329261472231. doi: 10.1177/11786329261472231 (PMC13400921; doi:10.1177/11786329261472231)
Supplement: Supplemental material - Adapting a Smartphone-Based Auricular Point Acupressure Self-Management Program for Rural Chronic Musculoskeletal Pain: Qualitative Study [file sj-pdf-2-his-10.1177_11786329261472231.pdf]

Supplementary File 2. Semistructured Focus Group Interview Guide

Question 1: How should we best refine (improve, adapt) the Auricular Point Acupressure Self-Management (APA-SM) program to better meet the needs of rural populations?

• Important factors to consider when adapting the intervention for rural populations

• How to make the program more culturally and linguistically appropriate

• How to improve relevance for Spanish-speaking populations and other diverse groups

• What program features, materials, or support would make APA-SM easier to understand and use

Question 2: How can we best integrate the APA-SM program into rural pain care?

• How best to recruit participants, including diverse populations

• Implementation strategies that may be most effective in rural communities

• Barriers and facilitators to implementation

• Things to consider to improve relevance for Spanish-speaking and other diverse populations

• How APA-SM could be integrated into existing care pathways or community settings

(Additional probes for non-patient stakeholders)

• How APA-SM could fit into current clinic or organizational workflows

• Where enrollment, introduction of the program, APA kit distribution, or follow-up could occur

• Perceived advantages of the intervention, including compatibility, importance, ease of use, and innovation

• Presence of clinic or organizational champions who could support implementation

• Implementation climate, work environment, leadership support, and relationships among administrators and staff

• Resources needed to support implementation, such as staffing, time, training, or technology access
